# Factors related to the experience of menopausal symptoms in women prescribed tamoxifen

**DOI:** 10.1080/0167482X.2016.1216963

**Published:** 2016-09-01

**Authors:** Zoe Moon, Myra S. Hunter, Rona Moss-Morris, Lyndsay Dawn Hughes

**Affiliations:** ^a^ Health Psychology Section, Institute of Psychiatry, Psychology & Neuroscience, King's College LondonLondonUK

**Keywords:** Health psychology, menopause, psycho-oncology

## Abstract

**Introduction:** Menopausal symptoms are frequent and severe in breast cancer survivors taking tamoxifen; however, treatment options are limited for these patients as hormonal replacement therapy is contraindicated. This study aimed to explore the experience and attribution of menopausal symptoms and identify factors related to the experience of menopausal symptoms in women taking tamoxifen.

**Methods:** Women who had been prescribed tamoxifen for a diagnosis of primary breast cancer were recruited from oncology clinics across England and from online advertisements. Seven hundred and forty women completed questionnaires assessing illness perceptions, social support, mood and symptom duration/severity.

**Results:** Eighty-four percent of women had experienced hot flushes and 80% experienced night sweats; of these, 60% experienced severe symptoms. Symptoms persisted throughout 5 years of treatment and were mainly attributed to tamoxifen. Logistic regressions showed that depressive symptoms, previous chemotherapy and being employed were associated with increased odds of hot flush or night sweat prevalence. Symptom severity was associated with depression, being employed and attributing symptoms to tamoxifen.

**Discussion:** These findings have clinical implications in terms of targeting women who are more at risk and offering non-hormonal treatment options, such as cognitive behavioural therapy, to help women to develop self-management strategies for coping with menopausal symptoms.

## Introduction

Hot flushes and night sweats (HFNS), the main symptom of the menopause, typically involve a sudden sensation of heat and warmth, accompanied by reddening of the skin and sweating. They are thought to result from disturbances of the temperature regulating mechanism in the hypothalamus, triggered by reduced oestrogen levels [1]. Whilst HFNS can vary significantly between individuals, women with breast cancer are five times more likely than age matched controls to experience these symptoms and are also more likely to experience longer, more frequent and more severe HFNS [2–4]. Women who take tamoxifen are twice as likely to experience HFNS [2] and more likely to report severe to intolerable HFNS [5] than other breast cancer survivors.

Tamoxifen, or a similar class of drugs (aromatase inhibitors), are prescribed to up to three quarters of breast cancer survivors in order to reduce the risk of recurrence [6]. They are prescribed to women with oestrogen receptor positive breast cancer and work by blocking the effects of oestrogen on cancer cells. Tamoxifen is prescribed mainly to pre-menopausal women, whereas aromatase inhibitors are prescribed only in post-menopausal women. Recent evidence suggests that survival benefits are enhanced if tamoxifen is taken for an additional 5 years [7,8]. This increase in treatment duration, accompanied by a rise in breast cancer survival rates, means that increasing numbers of women may be suffering from HFNS as a consequence of tamoxifen. Studies have indicated that HFNS prevalence in breast cancer survivors may be as high as 80% [9–11]. Tamoxifen is associated with a range of other side effects including weight gain, insomnia, joint pain and vaginal dryness [12,13]. Whilst not life threatening, these symptoms can have a considerable impact on quality of life [11]. HFNS in breast cancer survivors are associated with anxiety, sleep problems, poor emotional functioning [10] and poor physical health [14]. Furthermore, these symptoms can undermine adherence to tamoxifen [15,16].

One of the key treatments for HFNS, hormone replacement therapy (HRT) [17], is contraindicated in breast cancer survivors due to a potential increased risk of cancer recurrence, which severely limits treatment options for HFNS in these patients. There are some non-hormonal options, such as venlafaxine or gabapentin [18], but many breast cancer survivors are keen to avoid additional medications which likely have side effects [10]. Several recent papers have called attention to the lack of research into HFNS in breast cancer survivors [1,19] and highlighted a need to understand the experiences of these women, with a view to identifying safe and effective treatments [10,19,20].

Factors associated with HFNS in the general population include lower levels of education [21,22], African American race [23,24], younger age [25] and being without a partner [26,27]. The cognitive model of HFNS explains how the perception, attribution and appraisal of menopausal symptoms are influenced by cognitive factors, beliefs and mood [28]. For example, stress or negative affect can reduce the threshold for detection of physical sensations, and increase the likelihood that women will attend to, and therefore report, HFNS [28,29]. Anxiety has been shown to precede hot flushes [30]; however, studies suggest that there is a complex bi-directional relationship between HFNS and depression whereby HFNS can cause depressed mood, but may also be a result of depression [1,28,31].

Moreover, anxiety and depression are associated with negative beliefs, which in turn affect cognitive appraisal of symptoms [28]. For example, negative thoughts such as embarrassment, disgust and worry are linked to more problematic hot flushes [32]. The common sense model of illness representations posits that how patients represent symptoms and where they attribute them will likely guide how they cope with the symptom [33]. This may influence emotional reactions, illness outcomes and health behaviours such as treatment adherence or help seeking [34–37]. The cognitive model of HFNS has informed the development of cognitive behavioural therapy (CBT) for HFNS, which has been shown to reduce the impact of HFNS [38,39].

Whilst the cognitive model of HFNS is well accepted in the general population [1,40], the experience of menopausal symptoms in women taking tamoxifen remains under-researched. This is important considering the increasing rates of breast cancer, partnered with greater survivorship and increased duration of tamoxifen treatment. This paper aimed to explore the experience and attribution of menopausal symptoms in women prescribed tamoxifen and, using the cognitive model and other sociodemographic predictors, identify factors related to the experience of HFNS.

## Methods

The study was approved by the Northampton National Research Ethics Committee (Ref.: 14/EM/1207), with site specific approvals for each site.

### Participants and procedure

Participants were recruited through oncology clinics in 27 NHS Trusts across England and through advertisements on Facebook groups, Twitter and charity websites between April 2015 and October 2015. To be eligible for the study, patients had to be female, over 18, have a diagnosis of primary breast cancer and currently being prescribed tamoxifen. Women were screened in clinic and those who were eligible were invited to participate in the study either in the clinic or with a postal invitation. Women who replied to the online advert were screened by the researcher. Informed consent was taken from all participants. The questionnaire took approximately 15–20 min to complete; participants could complete it in clinic or online, or take it away and return it to the researcher using a stamped addressed envelope. This formed part of a larger study investigating adherence to tamoxifen. Only measures relevant to this study are reported here.

### Measures

#### Experience of menopausal symptoms

Participants were asked to indicate whether they had experienced symptoms using the identity scale from the Revised Illness Perceptions Questionnaire (IPQ-R [41]). This included the core symptoms from the IPQ-R as well as additional symptoms such as HFNS. Participants indicated whether they attributed symptoms to their breast cancer, their tamoxifen treatment or to previous cancer treatment. The additional concerns subscale from the FACT-ES [42] was used to measure the experience and severity of side effects. The FACT-ES is a quality of life scale for breast cancer patients taking endocrine therapy, with good internal consistency and test–retest reliability [42]. Participants rated symptom severity on five-point scales, from “not at all” to “very much”.

#### Potential predictors

Women were asked to provide sociodemographic data including their date of birth, age they left full-time education, relationship status, employment status, menopausal status (at diagnosis), date first prescribed tamoxifen and previous chemotherapy. Menopausal status was defined as pre-menopausal, menopausal or post-menopausal.

#### Mood

The Hospital Anxiety and Depression Scale (HADS [43]) was used to measure depression and anxiety. Each item is scored on a scale of 0–3, with higher scores reflecting higher levels of depression and anxiety. The scale has good internal consistency in patients with breast cancer [44,45].

#### Social support

The Multidimensional Scale of Perceived Social Support [46] was used to measure perceived social support. The scale has demonstrated good internal and test–retest reliability [46] and has been used successfully to measure social support in patients with breast cancer [47,48].

### Statistical analysis

Statistical analyses were performed using SPSS v21 (SPSS Inc., Chicago, IL). For analysis of symptom prevalence, women were coded as experiencing a symptom if they had selected answers on the FACT-ES from *a little bit* to *very much.* For analysis of symptom severity, women who scored either of the top two answers (*quite a bit/very much*) were coded as experiencing severe symptoms and were compared to women experiencing mild to moderate symptoms (*a little bit/somewhat*). The attribution of symptoms was analysed using responses on the IPQ-R. Univariate logistic regressions were calculated to assess the relationships between predictor variables and HFNS prevalence. Predictor variables were chosen based on the cognitive model and previous literature identifying sociodemographic variables which may be related to HFNS. Variables tested in univariate analysis were age, ethnicity, age left full time education, relationship status, employment status, menopausal status (at diagnosis), chemotherapy, months since first prescribed tamoxifen, anxiety, depression, social support and whether symptoms were attributed to tamoxifen. Months since first tamoxifen prescription, social support and depression were skewed and log transformations were performed. Variables which showed a significant relationship in univariate analysis were entered into a final multivariate model. Categorical variables such as ethnicity were converted into dichotomous dummy coded variables. The same analysis was then conducted to predict experience of severe HFNS in subgroup analyses of participants who had experienced these symptoms.

## Results

### Participant rate

One thousand two hundred and twenty-eight women were posted information about the study or approached in clinic. Seven hundred and forty-six women from 27 centres across England returned the questionnaire, giving a response rate of 61%. An additional six questionnaires were received from a site with no response rate information. Sixty-one women were recruited online. Once women who had reported discontinuing tamoxifen were removed (*n* = 73), the sample consisted of 740 women.

### Sample characteristics

The mean age was 53 (SD =10, range 30–90) (Table 1). Women were diagnosed with stage I to stage III breast cancer and were prescribed tamoxifen. The majority of participants were married/cohabiting (72%) and were employed (66%). Forty-nine percent left full time education under the age of 18. Over half of women were pre-menopausal at diagnosis (55%) and had been treated with chemotherapy (52%). Women had been taking tamoxifen for on average 20 months (SD = 18, range 0.2 months to 10 years).

**Table 1.** Demographics of study population.

	*N* (%)
Age, mean (SD)	53 (10)
	Range 30–90
Ethnicity	
White British	681 (92%)
Mixed/multiple ethnic	7 (1%)
Asian/Asian British	30 (4%)
Black/Black British	12 (2%)
Other ethnic background	10 (1%)
Relationship status	
Single	791 (11%)
Married	431 (58%)
Widowed	34 (5%)
Separated/divorced	91 (12%)
Co-habiting	102 (14%)
Employment status	
Employed full time	281 (38%)
Employed part time	204 (28%)
Homemaker	52 (7%)
Unemployed	57 (8%)
Retired	114 (15%)
Other	30 (4%)
Age left full time education	
Under 18	366 (49%)
Over 18	376 (51%)
Menopausal status at diagnosis	
Pre-menopausal	405 (55%)
Peri-menopausal	83 (11%)
Post-menopausal	202 (27%)
Unsure/missing	50 (7%)
Months since prescribed tamoxifen, mean (SD)	19.5 (18.3)
	Range 0.2–121
Received chemotherapy	381 (52%)

#### Experience, attribution and duration of menopausal symptoms

**Figure 1.**
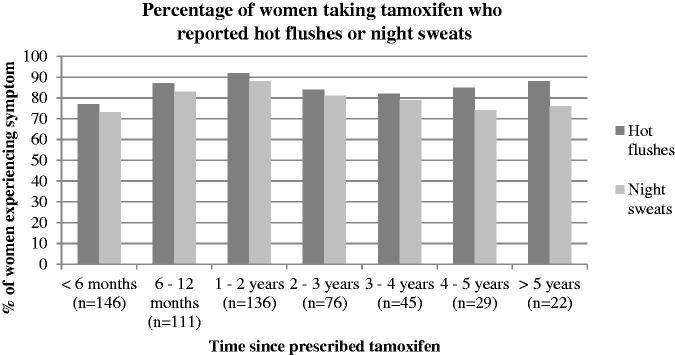
Percentage of women taking tamoxifen who reported hot flushes or night sweats.

**Figure 2.**
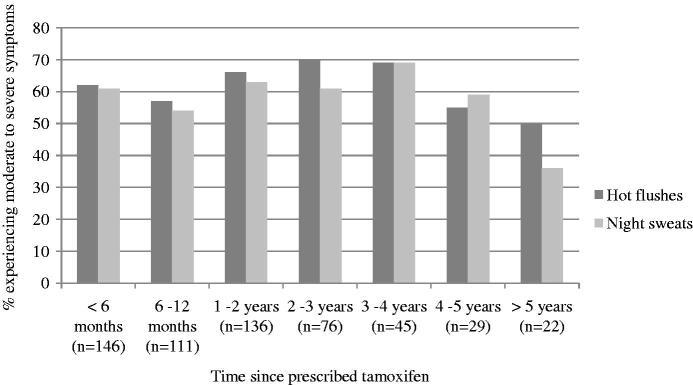
Percentage of women taking tamoxifen who reported severe hot flushes or night sweats.

A high percentage of participants had experienced hot flushes (84%) and/or night sweats (80%) and around 60% of these had experienced severe HFNS (Table 2). Patients also self-reported experiencing the following symptoms from the FACT-ES; fatigue (53%), weight gain (66%), mood swings (67%), loss of libido (68%), vaginal dryness/discharge/itchiness (72%) and joint pain (72%). All symptoms were attributed to tamoxifen more often than to breast cancer or previous cancer treatment. The symptoms most commonly attributed to tamoxifen on the IPQ-R were hot flushes (66%), night sweats (54%), weight loss/gain (40%), joint pain (37%), fatigue (35%), sleep difficulties (34%), vaginal dryness/discharge/itchiness (34%) and change in sex drive (27%). Figure 1 shows that the prevalence of HFNS is high across participants at different time points of treatment, including those in their fifth year. In separate analyses of those who had experienced symptoms (*n* = 623 for HF/*n* = 587 for NS), the proportion of women experiencing severe symptoms remains relatively high across the 5 years, but begins to decrease slightly at 4 years of treatment (Figure 2).

**Table 2.** Experience and attribution of symptoms.

	FACT-ES		IPQ-R		
	% Experienced inpast seven days	% With moderate to severe symptoms	% Attributed tobreast cancer	% Attributed to previous breast cancer treatment	% Attributed to tamoxifen treatment
Hot flushes	84	64	9	8	66
Night sweats	80	60	7	7	54
Change in sex drive^a^	40	–	17	8	27
Loss of sex drive	68	46	–	–	–
Pain or discomfort with intercourse	41	40	–	–	–
Vaginal discharge/dryness/itchiness	72	39	5	5	34
Weight gain	66	46			
Weight loss/gain^a^	47	–	10	10	40
Feeling down^a^	37	–	18	8	20
Mood swings	67	30		–	–
Fatigue^a^	53	–	19	13	35
Sleep difficulties^a^	44	–	13	9	34
Joint pain	72	55	6	14	37
Headaches	53	21	3	8	15
Loss of concentration^a^	38	–	12	9	24

Not all women who reported a symptom will have reported how they attributed it, and women could select multiple sources of attribution. % with moderate to severe symptoms in separate analysis of only those who experienced symptom.

^a^ These symptoms are not included in the FACT-ES and prevalence is derived from the IPQ-R.

#### Factors related to prevalence of HFNS

In the univariate analysis, younger age (OR = 0.95, 95% CI = 0.93–0.97), being employed (OR = 3.74, 95% CI = 2.45–5.72), being premenopausal at diagnosis (OR = 1.95, 95% CI = 1.29–2.94), receiving chemotherapy (OR = 3.13, 95% CI = 2.04–4.80) and having higher levels of anxiety (OR = 1.09, 95% CI =1.04–1.15) and depression (OR = 1.90, 95% CI = 1.24–2.90) were significantly related to hot flush experience (Table 3). These variables were entered into a logistic regression model, which explained 15% of the total variance (Nagelkerke R^2^). Women who were employed (OR = 2.65, 95% CI = 1.44–4.90), who scored higher on the HADS depression scale (OR = 2.22, 95% CI = 1.33–3.70) and who had chemotherapy (OR = 1.93, 95% CI = 1.14–3.26) were around twice as likely to experience hot flushes (Table 4).

**Table 3.** Univariate regressions predicting prevalence of HFNS.

	Hot flushes		Night sweats	
	OR	95% CI	OR	95% CI
Age	0.95**	0.93–0.97	0.97**	0.95–0.98
Ethnicity				
Other versus White British	1.18	0.68–2.06	1.16	0.70–1.92
Age left full time education				
<18 versus 18+	1.05	0.71–1.55	1.00	0.70–1.42
Employment status				
Employed versus not employed	3.74**	2.45–5.72	2.41**	1.63–3.56
Marital status				
No partner versus partner	0.80	0.52–1.23	0.63*	0.43–0.92
Menopausal status				
Pre- versus post-menopausal	1.95*	1.29–2.94	1.46*	1.00–2.11
Chemotherapy	3.13**	2.04–4.80	1.91**	1.32–2.74
Months since prescribed	1.10	0.99–2.23	1.03	0.94–1.13
HADS anxiety	1.09*	1.04–1.15	1.11**	1.06–1.16
HADS depression	1.90*	1.24–2.90	2.42**	1.51–3.32
Social support	1.35	0.97–1.89	1.15	0.85–1.54

** *p* < (0).001.

* *p* < 0.05.

**Table 4.** Multivariate regressions predicting prevalence/severity of HFNS.

	Hot flushes prevalence		Night sweat prevalence		Hot flush severity		Night sweat severity	
	OR	95% CI	OR	95% CI	OR	95% CI	OR	95% CI
Employment status (employed versus not employed)	2.65*	1.44–4.90	2.18*	1.24–3.82	1.68*	1.03–2.73		
Chemotherapy	1.93*	1.14–3.26						
HADS depression	2.22*	1.33–3.70	2.41*	1.34–4.33	1.99*	1.22–3.24	1.10**	1.03–1.17
Attributing HF/NS to tamoxifen					3.78**	2.43–5.77	2.80**	1.94–4.01

** *p* < 0.001.

* *p* < 0.05.

In the univariate analysis (Table 3), experience of night sweats was related to younger age (OR = 0.97, 95% CI = 0.95–0.98), being employed (OR = 2.41, 95% CI = 1.63–3.56), being premenopausal (OR = 1.46, 95% CI = 1.00–2.11), being without a partner (OR = 0.63, 95% CI = 0.43–0.92), receiving chemotherapy (OR = 1.91, 95% CI = 1.32–2.74) and higher levels of anxiety (OR = 1.11, 95% CI = 1.06–1.16) and depression (OR = 2.42, 95% CI = 1.51–3.32). These variables were entered into a logistic regression model which accounted for 12% of the total variance; women with more depressive symptoms (OR = 2.41, 95% CI = 1.34–4.33) and who were employed (OR = 2.18. 95% CI = 1.24–3.82) were more likely to experience night sweats (Table 4).

#### Factors related to severity of HFNS

In the univariate analysis of those who experienced hot flushes (*n* = 623), hot flush severity was associated with being employed (OR = 1.62, 95% CI = 1.07–2.43), premenopausal (OR = 1.52, 95% CI = 1.07–2.14), having chemotherapy (OR = 1.56, 95% CI = 1.12–2.17), higher levels of anxiety (OR = 1.07, 95% CI = 1.03–1.11) and depression (OR = 2.04, 95% CI = 1.45–2.87) and attributing hot flushes to tamoxifen (OR = 2.58, 95% CI = 1.77–3.77) (Table 5). Variables were entered into a final model which explained 18% of the variance in hot flush severity (Table 4). Women who attributed their hot flushes to tamoxifen were almost four times more likely to experience more severe hot flushes (OR = 3.78, 95% CI = 2.43–5.77) and women who had more depressive symptoms (OR = 1.99, 95% CI = 1.22–3.24) or were employed (OR = 1.68, 95% CI = 1.03–2.73) were almost twice as likely to experience severe hot flushes.

**Table 5.** Univariate regressions predicting severity of hot flushes (*n* = 623) and night sweats (*n* = 587).

	Hot flushes		Night sweats	
	OR	95% CI	OR	95% CI
Age	0.98	0.97–1.00	0.98	0.97–1.00
Ethnicity				
Other versus white British	1.24	0.77–1.99	1.39	0.86–2.24
Age left full time education				
<18 versus 18+	1.07	0.77–1.48	1.28	0.92–1.78
Employment status				
Employed versus not employed	1.62**	1.07–2.43	1.23	0.81–1.87
Marital status				
No partner versus partner	0.87	0.60–1.25	0.86	0.59–1.25
Menopausal status				
Pre versus post-menopausal	1.52*	1.07–2.14	1.31	0.92–1.85
Chemotherapy	1.56*	1.12–2.17	1.06	0.76–1.47
Months since prescribed	1.02	0.94–1.12	1.00	0.92–1.09
HADS anxiety	1.07*	1.03–1.11	1.06**	1.02–1.11
HADS depression	2.04**	1.45–2.87	2.03**	1.46–2.83
Social support	1.56	0.93–2.62	1.14	0.69–1.91
Symptom attributed to tamoxifen	2.58**	1.77–3.77	2.63**	1.84–3.74

** *p* < 0.001.

* *p* < 0.05.

In the univariate analysis of participants who experienced night sweats (*n* = 587), anxiety (OR = 1.06, 95% CI = 1.02–1.11), depression (OR = 2.03, 95% CI = 1.46–2.83) and attribution of night sweats to tamoxifen (OR = 2.63, 95% CI = 1.84–3.74) were related to night sweat severity. All variables except anxiety remained significant in the multivariate analysis, accounting for 11% of the total variance (Table 4). Attributing night sweats to tamoxifen (OR = 2.80, 95% CI = 1.94–4.01) and depression (OR = 1.10, 95% CI = 1.03–1.17) were both linked to increased odds of severe night sweats.

## Discussion

This paper examined the experience of menopausal symptoms in breast cancer survivors taking tamoxifen and explored factors contributing to the experience of HFNS. Results showed that 84% of women had experienced hot flushes and 80% had experienced night sweats. This is consistent with previous research in the community indicating a prevalence of around 80% [9–11], but is much higher than the prevalence of 29–45% found in several large RCTs comparing tamoxifen with aromatase inhibitors [49]. This may be because some women who experienced negative side effects discontinued treatment and were removed from the RCTs. However, previously, less was known regarding the severity of HFNS in women taking tamoxifen [50]. This paper adds new information, by showing that around 60% of women experiencing HFNS reported severe symptoms. The extent and severity of these symptoms reinforces the need to identify who is more at risk and to find ways to help patients manage these symptoms [1,19,20]. Participants also reported high levels of joint pain, vaginal discharge/dryness/itchiness, loss of libido, mood swings and weight gain. The prevalence of fatigue and sleep problems was slightly lower than previously reported in patients taking tamoxifen [13,51], but loss of libido, vaginal symptoms and mood swings were higher than previous reports have indicated [13,52,53]. Again, all symptoms were reported at a greater frequency than found in a review of RCTs [49].

Previous studies have suggested that HFNS are less problematic after one year of tamoxifen treatment [54,55] and patients are often advised that their symptoms will reduce after a few months. However, this study shows that the prevalence of HFNS remains stable (around 80%) regardless of whether the patient is in her first or fifth year of treatment. The severity of symptoms also remains high up until the fourth year of treatment. This highlights the need to identify effective strategies to help women to manage their HFNS across the duration of treatment. CBT has been shown to reduce HFNS frequency and problem rating in breast cancer survivors and can teach women long-term self-management strategies [38,39,56].

Up to two-thirds of participants attributed HFNS to tamoxifen. Participants also associated other symptoms to tamoxifen, including fatigue, sleep difficulties, joint pain, vaginal discharge/dryness/itchiness and weight loss/gain. These symptoms are established side effects of tamoxifen [13]. Women who attributed HFNS to tamoxifen were three to four times more likely to experience severe symptoms than those who did not attribute their symptoms to tamoxifen. More research is needed to confirm the direction of this effect and to establish the consequences of attributing symptoms to tamoxifen treatment. Previous studies have suggested that symptom attribution is likely to affect coping behaviours [33], but this was not tested in the current study.

After controlling for demographic factors and mood, women who had chemotherapy were twice as likely to report hot flushes than women who had not had chemotherapy. This conflicts with previous studies in breast cancer patients, showing no association between HFNS and chemotherapy [11,57]. However, previous studies included mainly postmenopausal women, and the association between chemotherapy and HFNS may be stronger in premenopausal women [58]. Chemotherapy can induce an early menopause in some patients, increasing the incidence of HFNS [59], which could explain the increased HFNS in premenopausal women who have received chemotherapy.

Women who were employed were twice as likely to experience HFNS and more likely to experience severe hot flushes. This has important implications for supporting women in the workplace. Studies have shown that menopausal symptoms cause difficulty at work and may impact negatively on work performance [1,60,61]. Working women have discussed fears around embarrassment and others’ reactions [62], which is likely to exacerbate the severity of hot flushes. CBT may be helpful to moderate negative thoughts around menopausal symptoms in the workplace and to reduce anxiety around stigma.

Higher scores on the depression scale were associated with twofold increased odds of HFNS incidence and increased odds of severe HFNS. This supports the cognitive model of HFNS [28], which proposes that depressed mood can affect how patients perceive and appraise their symptoms. However, it is likely that there is a bi-directional relationship between HFNS and depression, and it is unclear in this study if the depressed mood is a result of the HFNS or if it is increasing the likelihood that women will report symptoms. Anxiety was associated with increased odds of HFNS in the univariate analysis, but was not significant after controlling for other variables. This contrasts with previous studies showing a clear relationship between anxiety and hot flushes [30]. However, the lack of relationship between anxiety and HFNS has been shown previously in breast cancer patients [63].

Age was significantly related to HFNS prevalence in the univariate analysis, but was not significant in the multivariate analysis. This is likely due to shared variance between age and menopausal status at diagnosis. Younger age has been found to be associated with increased risk of hot flushes in breast cancer survivors [5]; however, this effect has not been consistently shown [11,13]. Previous studies have shown that ethnicity is related to hot flush frequency [64]. African-American women tend to report more hot flushes than Caucasian women and Japanese women have been shown to report fewer symptoms [24,65,66]. However, these effects are not always shown [67] and the current study found no effect of ethnicity on HFNS prevalence or severity. This may be due to the lack of ethnic diversity in the study; only 8% of women self-identified as not White British.

Overall, the results suggest that a high proportion of women experience symptoms such as HFNS as well as fatigue, joint pain and vaginal symptoms. These symptoms are often severe and women report experiencing them even in their fifth year of treatment. As HRT is contraindicated, only 21% of breast cancer survivors receive any treatment for these symptoms [11] and there is a need to identify non-hormonal treatments. The North American Menopause Society (NAMS) has reviewed evidence for non-hormonal treatments and has found some degree of efficacy for selective serotonin reuptake inhibitors in menopausal women [68], but results are not conclusive and breast cancer survivors have expressed a preference for non-medical treatments [10]. CBT, which is based on the cognitive model of HFNS, is recommended by NAMS [69] and The National Institute for Health and Care Excellence [17]. CBT has been shown to improve HFNS problem rating and may provide patients with long lasting self-management strategies. There is a need to identify patients who are taking tamoxifen and have received chemotherapy, as they may be more at risk of hot flushes. Furthermore, the results stress a need to support women who have returned to work following breast cancer.

The strengths of this study were the large sample size, use of validated measures and good response rate. This is one of the largest samples used to investigate the experience of HFNS in women taking tamoxifen. However, that we measured symptom severity and not bother is a limitation of this study. Measuring perceived bother from symptoms as opposed to the severity may provide a more thorough understanding of the impairment associated with these symptoms [66]. An additional limitation was the use of cross-sectional data which prohibits causal assumptions for some effects, such as the relationship between hot flushes and depression. All measurements were subjective; therefore, the hot flush frequency may be more of an assessment of how people perceive their symptoms rather than an objective physiological measure. Data were not collected on use of additional medications. Some women may be prescribed anti-depressants to manage their HFNS, and this could have impacted on their mood. A final limitation with the study was the lack of a comparison group, such as breast cancer patients not receiving endocrine therapy, with whom to compare the results to.

## Conclusion

Prevalence and severity of HFNS, as well as other symptoms such as vaginal dryness and joint pain, are high in breast cancer survivors taking tamoxifen. There is a need to identify non-hormonal treatment options such as CBT to help support patients with these symptoms, especially as they persist for longer than previously believed. Furthermore, this study shows that women who are in employment, received chemotherapy, attribute HFNS to tamoxifen and have high depression scores may require more targeted support to manage HFNS.

## Notes

**Current knowledge on this subject**
- Hot flushes and night sweats (HFNS) are common and severe in breast cancer survivors taking tamoxifen, yet HFNS treatment options for these patients are limited.
- Sociodemographic and psychological factors have been shown to be related to HFNS in the general population.
- Little is known about the experience of HFNS in breast cancer survivors taking tamoxifen, and few predictors of HFNS have been found.

**What this study adds**
- Over 80% of women taking tamoxifen report experiencing HFNS, and around 60% of these women report severe symptoms.
- Symptoms are mainly attributed to tamoxifen and the prevalence remains high even in the fourth and fifth years of treatment, challenging previous research suggesting that HFNS would lessen after the first year of treatment.
- Previous chemotherapy, higher levels of depression, being employed and attributing symptoms to tamoxifen are related to HFNS prevalence/severity in breast cancer survivors taking tamoxifen.
